# Natural History and Hepatitis B Virus Surface Antigen (HBsAg) Spontaneous Seroclearance in Hepatitis B Virus e-Antigen (HBeAg)-Negative Patients with Inactive Chronic Infection: A Multicenter Regional Study from South Italy

**DOI:** 10.3390/pathogens12101198

**Published:** 2023-09-26

**Authors:** Michele Barone, Andrea Iannone, Martino Mezzapesa, Michele Milella, Francesco Di Gennaro, Grazia Niro, Rosa Cotugno, Raffaele Cozzolongo, Giuseppe Mennea, Maria Rendina, Alfredo Di Leo

**Affiliations:** 1 Gastroenterology Unit, Department of Precision and Regenerative Medicine—Jonian Area—(DiMePRe-J), University of Bari “Aldo Moro”, Policlinic University Hospital, 70124 Bari, Italy; alfredo.dileo@uniba.it; 2 Gastroenterology Unit, Policlinic University Hospital, Piazza G. Cesare 11, 70124 Bari, Italy; ianan@hotmai.it (A.I.); martino.mezzapesa@gmail.com (M.M.); mariarendina@virgilio.it (M.R.); 3 Clinic of Infectious Diseases, Policlinic University Hospital, 70124 Bari, Italy; michele.milella@tin.it; 4 Clinic of Infectious Diseases, Department of Precision and Regenerative Medicine—Jonian Area—(DiMePRe-J), University of Bari “Aldo Moro”, Policlinic University Hospital, Piazza G. Cesare 11, 70124 Bari, Italy; francesco.digennaro1@uniba.it; 5 Division of Gastroenterology and Endoscopy, Fondazione IRCCS ‘Casa Sollievo della Sofferenza’, Viale Cappuccini, snc, 71013 San Giovanni Rotondo, Italy; g.niro@operapadrepio.it (G.N.); r.cotugno@operapadrepio.it (R.C.); 6 Gastroenterology Unit, IRCCS “S. De Bellis”, Via Turi 27, 70013 Castellana Grotte, Italy; raffaele.cozzolongo@irccsdebellis.it; 7 Internal Medicine Unit, “L. Bonomo” Hospital, Viale Istria, 76123 Andria, Italy; giuseppe.mennea@aslbat.it

**Keywords:** HBV, chronic infection, HBsAg seroclearance, inactive carrier

## Abstract

Spontaneous HBsAg seroclearance has been mainly studied in populations from Asia, Australia, the Pacific Islands, and Polynesia. For the first time, we evaluated the spontaneous HBsAg seroclearance and its possible associated factors and the risk of disease progression in HBeAg-negative patients with inactive infection all coming from the same region in South Italy. In this multicenter retrospective study, 146 patients were selected after 18 months of observation and followed for a median of 82 months (IQR 60–107). For our analyses, they were divided into three groups based on their HBsAg levels: <100 IU/mL, 100–1000 IU/mL, and >1000 IU/mL. Crude and adjusted hazard ratios (HRs) for HBsAg seroclearance were determined. During the follow-up period, three patients (2.0%) showed a disease progression with an increased liver stiffness, whereas 17 (11.6%) cleared the HBsAg. Patients with HBsAg levels <100 IU/mL had the highest probability of HBsAg seroclearance compared to the other two groups (*p* = 0.009). In the multivariate analysis, the HBsAg level <100 IU/mL was the only parameter independently associated with HBsAg seroclearance (adjusted HR = 3.53; CI 1.29–9.69; *p* = 0.01). In patients with chronic HBV inactive infection, HBsAg levels <100 IU/mL predicted the highest probability of HBsAg seroclearance.

## 1. Introduction

The clearance of HBV-DNA and HBsAg in the serum associated with the appearance of anti-HBs antibodies (seroconversion) is considered to be the final step in healing from an HBV infection. However, the simple HBsAg disappearance from the serum represents an ideal end point since it indicates profound suppression of the HBV replication and viral protein expression [1]. 

The spontaneous annual HBsAg seroclearance rate in patients with chronic HBV infection varies from 0.54% to 1.98% in Western cohorts [2] and is 1.3% (95% CI, 1.25–1.38) in cohorts from Asia, the Pacific Islands, and Polynesia [3].

The highest spontaneous HBsAg seroclearance rate has been observed in subjects with a chronic inactive HBV infection defined as HBeAg-negativity, undetectable or low HBV DNA (<2000 IU/mL or in some cases, <20,000 IU/mL), persistently normal ALT, and minimal hepatic fibrosis and necroinflammatory activity [1]. Several studies have investigated the viral and host characteristics in the different phases of the natural history of the diseases in patients that undergo spontaneous seroclearance [2]. 

Brunetto et al. [4] were the first who, using a cohort of 209 HBV genotype D carriers, identified the “inactive carriers” (ICs) state with 91.1% sensitivity, 95.4% specificity, 87.9% positive predictive value, 96.7% negative predictive value, using a combined single point quantification of HBsAg <1000 IU/mL and HBVDNA ≤2000 IU/mL. This achievement required prospectively monthly serum test evaluations during the first 12 months of the study, which continued later on every 3 months together with an annual determination of liver stiffness by transient elastography. A successive retrospective study by Brouwer et al. [5] on 292 HBV carriers with genotypes A-D coming from Europe, Asia, and Australia reported that ICs with quantitative HBsAg serum levels <100 IU/mL and HBV DNA level <2000 IU/mL would have the highest probability of spontaneous HBsAg seroclearance at 5 and 10 years (cumulative incidence rates of 39.1% and 63.3%, respectively). A more recent retrospective study on a large cohort of HBV carriers at different phases of chronic HBV infection [3], coming from Asian, Pacific Islander, and Polynesian centers, added other predictors for spontaneous HBsAg seroclearance such as older age, male sex, and genotype C vs. genotype B. In this study, the authors observed an annual seroclearance rate of 1.31% in all patients, which became over 7% in subjects with HBVDNA <2000 IU/mL, normal ALT (defined as “immune inactive”) aged >55 years, and with HBsAg <100 IU/mL, whereas the cumulative incidence rate of spontaneous HBsAg seroclearance at 5 and 10 years reached 22.5% and 39.1% when they considered all subjects with HBsAg < 100 IU/mL. The genotype influence on spontaneous HBV seroclearance [3,6] and antiviral therapy [6,7] suggests a different natural history of the infection in the various geographical areas. For this reason, we evaluated both the rate of spontaneous HBsAg seroclearance and its possible predictors in Caucasian subjects coming from the same region of South Italy who were affected by a chronic inactive HBV infection. In addition, we also evaluated the risk of disease progression since a low risk of cirrhosis or HCC persists in these patients [1,8].

## 2. Materials and Methods

### 2.1. Study Design

We performed a multicentric retrospective study in HBeAg-negative patients, aged > 18 years, with chronic HBV infection who attended three tertiary care and one primary care center from June 2010 to February 2023. All our patients were Caucasian, 143 were Italian, 2 were from East Europe, and 1 was from Albania. The study was conducted in accordance with the Declaration of Helsinki (2000). All participants gave informed consent, allowing their anonymized information to be used for data analysis. The research protocol was approved by the local Ethics Committee (N.674/DG). 

Patients underwent 6-month monitoring for 18 months, during which we assessed HBsAg positivity or quantitative HBsAg (qHBsAg), liver chemistry, and HBV-DNA. Among them, we selected only subjects who were affected by an inactive infection (HBV DNA ranging from <2000 IU/mL and <20,000 IU/mL, persistently normal ALT, and absent or minimal hepatic fibrosis assessed by transient elastography) [1]. For our analyses, we divided the selected patients into three groups based on their HBsAg levels: <100 IU/mL, 100–1000 IU/mL, and >1000 IU/mL. 

We excluded patients coinfected with either HCV, HDV, HIV, or a medical history of tumors or transplantation, or those undergoing immunosuppressive or biologic therapy; we also excluded patients with other known liver diseases.

The primary outcome of this study was the achievement of spontaneous HBsAg seroclearance, defined as having ≥2 negative HBsAg results ≥6 months apart in the absence of any antiviral treatment. The secondary outcome was to evaluate the factors associated with spontaneous HBsAg seroclearance. Patients’ censoring occurred once they reached HBsAg seroclearance, possible reactivation, death, and end of follow-up. The follow-up length went from the date of inclusion to the last visit.

### 2.2. Laboratory Data and Transient Elastography

Serum HBV DNA levels were quantified by using the COBAS TaqMan assay, with sensitivity 12 IU/mL and dynamic range 6.0–1.10 × 10^8^ IU/mL. From the beginning of the study, we used the Architect platform (Abbott Laboratories, Abbott Park, IL, USA), with a detection limit of 0.05 IU/mL, to assess HBsAg serum levels. We used standard laboratory methods for serum liver enzymes determinations. Antibodies to HBsAg, hepatitis C, hepatitis D, and human immunodeficiency virus were detected by using commercially available immunoassays. All subjects underwent the determination of liver stiffness by a FibroScan apparatus (Echosens, Paris, France) after an overnight fasting period. We used a cut-off value < 6 kPa (F0) to exclude the presence of fibrosis as previously suggested [4].

### 2.3. Statistical Analysis

Categorical variables were expressed as counts and percentages. Continuous variables were reported as mean with standard deviation (SD) or median with interquartile range (IQR), according to their distribution assessed by the Shapiro–Wilk test of normality. Among baseline HBsAg level groups, categorical data were compared using the χ^2^ test, while, for continuous parameters, we used the analysis of variance (ANOVA) or Kruskal–Wallis test, as appropriate. The Kaplan–Meier analysis estimated the cumulative spontaneous HBsAg seroclearance rates at 5 and 10 years according to the baseline HBsAg levels (i.e., <100 IU/mL, 100–1000 IU/mL, and >1000 IU/mL). The difference among these three groups was evaluated by the log-rank test. We also calculated the quartiles of the distribution of baseline HBsAg <100 IU/mL. Then, we used ROC analysis to estimate sensitivity and specificity for predicting HBsAg seroclearance and for deciding the cut-off point on the basis of the criterion that maximizes the Youden’s J index. The Kaplan–Meier method was used to assess the association between baseline HBsAg levels, sex, age, follow-up duration, body mass index (BMI), HBV-DNA levels, liver stiffness, and liver steatosis with spontaneous HBsAg seroclearance. Time to event was defined as the time from the diagnosis of chronic inactive HBV infection to HBsAg seroclearance or censoring. Patients were censored if they were event-free through the end of the study observation. The log-rank test was used to assess the effect of each variable on HBsAg seroclearance. Crude hazard ratios (HRs) with their 95% confidence intervals (95% CIs) were estimated by univariate Cox proportional hazards models. Variables showing a *p*-value < 0.20 from the univariate analysis were included in the baseline multivariate proportional hazards model. The final multivariate model was obtained by estimating the effect of each variable on the outcome using the backward method. Adjusted HRs and their 95% CIs were estimated. In the multivariate model, the proportional hazard assumption for each variable was assessed by fitting time-dependent covariates. Statistical significance was set at *p* < 0.05. All statistical analyses were performed using the Statistical Analysis Software (SAS Institute Inc., Cary, NC, USA).

## 3. Results

At the time when we started to collect data, a total of 1068 HBsAg-positive patients attended our four centers. Among them, 922 (86.3%) were receiving nucleot(s)ide therapy, and 146 (13.7%) had a chronic inactive HBV infection. Table 1 reports the baseline characteristics of the 146 patients that were enrolled in the study. We divided these patients into three groups according to baseline HBsAg levels.

The only parameters that was significantly different in addition to HBsAg levels were age (*p* < 0.001), BMI (*p* = 0.04), and HBV-DNA (*p* = 0.002). In particular, the group with HBsAg <100 IU/mL had the higher age, the higher BMI, and the lowest HBV-DNA levels.

**Figure 1:**
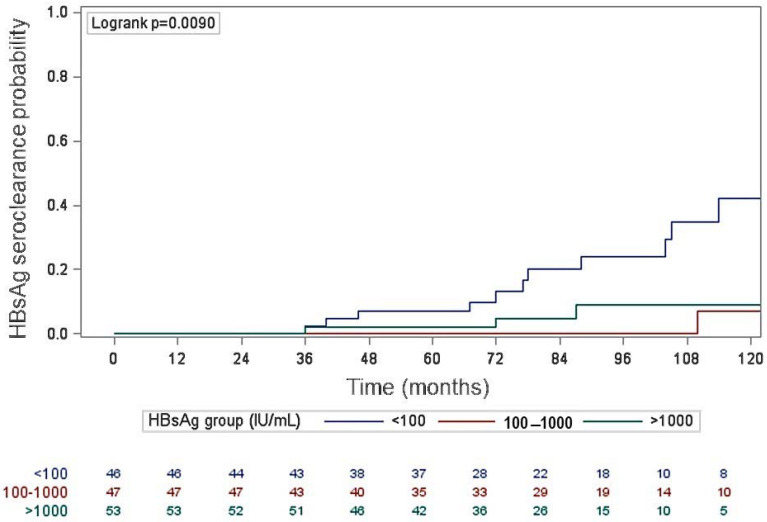
Probability of HBsAg seroclearance in the three groups of patients selected on the basis of HBsAg levels.

During the follow-up period of 10 years, among our 146 patients, 3 (2.0%) showed a liver stiffness increase from 5.2 ± 0.4 to 8.2 ± 0.5 (one of these three patients had HBsAg <100 and two had HBsAg >1000 IU/mL), and 17 (11.6%) cleared the HBsAg. We excluded other possible subsequent comorbidities in at least two of the three patients who experienced an increase in liver stiffness, probably related to undetected HBV flares. In the third patient, there was a significant increase in the BMI (from 21.6 to 28.6 kg/m^2^), probably responsible for a progression to steatohepatitis. The probability of HBsAg seroclearance was significantly higher for patients with HBsAg levels <100 IU/mL compared to the group with HBsAg levels >1000 IU/mL, whereas there was no difference between the latter group and patients with HBsAg levels ranging from 100 to 1000 IU/mL (Figure 1). We also tested the predictive value of HBsAg using the quartile distribution of baseline HBsAg <100 IU/m, confirming the best predictive value at HBsAg <100 IU/mL. As a matter of fact, the baseline HBsAg cut-off value of 100 IU/mL showed the highest Youden’s J index, with a sensitivity of 64.7% and a specificity of 72.9%.

When we calculated, in all patients, the 5- and 10-year cumulative incidence of HBsAg seroclearance, the value was 3.0% (CI 1.2–7.9%) and 17.4% (CI 10.8–27.6%), respectively (Table 2).

However, the cumulative incidence varied in the three groups, with the highest being in the HBsAg levels <100 IU/mL [7.0% (CI 2.3–20.1%) at 5 years and 35.0% (CI 20.9–54.8%) at 10 years] and decreasing in the HBsAg levels 100–1000 IU/mL [0% at 5 years and 4.4% (CI 0.6–27.1%) at 10 years] and the HBsAg levels > 1000 IU/mL [2.1% (CI 0.3–23.9%) at 5 years and 10.1% (CI 3.2–29.2%) at 10 years] (*p* = 0.009, by log-rank test) (Table 2). The annual rate of spontaneous HBsAg seroclearance in all 146 patients was 1.65% (95% CI = 1.05–2.25%).

Since there was no difference in HBsAg seroclearance probability between patients with HBsAg levels of 100–1000 IU/mL and > 1000 IU/mL, we combined these two groups to perform the analysis of HBsAg clearance predictors (Table 3). From the univariate analysis, patients with HBsAg <100 IU/mL had a higher probability of HBsAg clearance when compared to those ≥ 100 IU/mL (HR = 4.18; CI = 1.55–11.32; *p* = 0.002). Moreover, males had a higher probability of HBsAg seroclearance when compared to females (HR = 3.51; CI = 1.14–10.81; *p* = 0.02) (Table 3).

In the multivariate analysis using the Cox proportional hazards models, the baseline HBsAg level <100 IU/mL was the only parameter independently associated with HBsAg seroclearance (adjusted HR = 3.53; CI = 1.29–9.69; *p* = 0.01), whereas the male sex only approximated statistical significance (adjusted HR = 2.84; CI = 0.90–8.90; *p* = 0.06) (Table 3).

## 4. Discussion

The World Health Organization (WHO) has estimated that, in 2019, about 14 million persons were chronically infected with the hepatitis B virus in the European region [9]. Although Italy is considered a country with a low prevalence of HBV infection, the significant migration in recent years has increased the occurrence of the disease [10].

The great majority of HBV chronically infected patients in Europe is represented by hepatitis B “e” antigen-negative (HBeAg-) subjects [11,12]. Among them, those with a chronic HBV inactive infection have the most favorable long-term outcome, although they are considered at low risk of progression to cirrhosis or HCC [8].

For the present study, patients were identified as carriers with a chronic HBV inactive infection after a strict follow-up of 18 months. Then, they underwent annual monitoring of viral and host laboratory parameters, echotomography, and evaluation of liver stiffness by transient elastography. 

On the basis of the data in the literature [3,4,13], we divided patients into three groups according to HBsAg levels and compared the subjects with <100 IU/mL with those with 100–1000 and >1000 IU/mL. However, since some data of the literature suggested a cut-off lower than 100 IU/mL to predict seroclearance [14,15], we also tested the predictive value of HBsAg using the quartile distribution of baseline HBsAg <100 IU/m. This analysis confirmed that the best predictive value for HBsAg seroclearance was 100 IU/mL. Patients with HBsAg <100 IU/mL were significantly older and had lower HBV DNA levels when compared to the other two groups, confirming the literature data. In addition, they showed a significantly higher BMI. None of them, except three, underwent a progression of the disease, as suggested by the increase in liver stiffness, and, for this reason, we excluded them from our analysis. 

The cumulative HBV seroclearance rate was significantly higher for the <100 IU/mL group when compared to the other two groups that showed a similar rate. Surprisingly, in the former group, the cumulative HBV seroclearance at 5 years (7%) was much lower compared to that which was reported by Brower et al. (39.1%) [4] and Yeo et al. (22.48%) [2] in a similar setting of patients. However, our cumulative HBV seroclearance at 10 years (35.1%) and the annual rate of spontaneous HBsAg seroclearance (1.65%) became similar to those reported by Yeo et al. (39.1% and 2.22%, respectively). In the multivariate analysis, the HBsAg <100 IU/mL was the only parameter that remained a significant predictor of seroclearance. Older age approximated statistical significance (*p* = 0.06), whereas the male sex remained influential to seroclearance. The lack of agreement compared to the data of the literature could be due to the lower number of enrolled patients; moreover, the different geographical origins of the patients should be considered. The two previous studies included cohorts prevalently from Asia and Australia, as well as Asia, the Pacific Islands, and Polynesia, respectively, i.e., geographical areas where genotypes B and C are the most represented; this aspect seems important when considering that the adjusted hazard ratio of spontaneous HBsAg seroclearance is 1.71 for genotype C compared to genotype B [2]. 

The major limitation of our study is the lack of genotype assessment; however, all patients, except three from East Europe, were from the same area of South Italy and therefore were presumed to be mostly genotype D [16]. The limited number of patients did not allow us to perform sub-analyses in patients with HBsAg <100 IU/mL and HBV DNA ≤2000 IU/mL.

## 5. Conclusions

Considering that the survival of patients with inactive chronic HBV infection is comparable with the noninfected population, at least in Western countries [17,18], the identification of predictors that allows us to recognize a chronic inactive HBV infection state has evident advantages in terms of saving medical resources and improving the quality of life of patients.

## Figures and Tables

**Table 1:** Baseline characteristics of included patients according to baseline HBsAg levels.

		Baseline HBsAg Levels			
	Total (n = 146)	<100 IU/mL (n = 46)	100–1000 IU/mL (n = 47)	>1000 IU/mL (n = 53)	*p* Value
Age, mean ± SD, years	57.7 ± 11.0	62.3 ± 8.7	58.9 ± 9.5	52.7 ± 12.1	<0.0001
Male/Female, n	69/77	27/19	22/25	20/33	0.11
BMI, mean ± SD, Kg/m^2^ *	26.4 ± 4.6	27.8 ± 4.6	26.3 ± 4.4	25.1 ± 4.4	0.04
Follow-up, mean ± SD, months	87.1 ± 32.1	89.8 ± 33.3	88.5 ± 34.3	83.5 ± 29.0	0.58
HBsAg, median (IQR), IU/mL	425 (84–1682)	21 (10–80)	400 (201–527)	2243 (1500–5422)	n.a.
ALT, median (IQR), U/L	21 (18–29)	23 (18–29)	20 (18–28)	21 (17–28)	0.63
AST, median (IQR), U/L	20 (17–25)	21 (17–24)	20 (17–23)	22 (19–27)	0.21
GGT, median (IQR), U/L	19 (14–28)	21 (16–31)	17 (14–22)	22 (14–29)	0.33
HBV-DNA, median (IQR), IU/mL *	567 (143–1639)	211 (72–775)	706 (201–1785)	739 (270–4031)	0.002
FibroScan, median (IQR), kPa	4.8 (4.3–5.5)	4.7 (4.0–5.5)	4.9 (4.3–5.7)	4.7 (4.3–5.4)	0.76
Liver steatosis, n/N (%) *	47/113 (41.6)	14/32 (43.8)	16/34 (47.1)	17/47 (36.2)	0.59
Diabetes or hyperglycemia, n/N (%) *	22/113 (19.5)	9/32 (28.1)	6/34 (17.7)	7/47 (14.9)	0.33

SD, standard deviation; HBsAg, hepatitis B serum antigen; BMI, body mass index; IQR, interquartile range; ALT, alanine aminotransferase; AST, aspartate aminotransferase; GGT, gamma-glutamyltransferase. * Missing data: 32 patients for BMI; 33 patients for steatosis; 33 patients for diabetes or hyperglycemia.

**Table 2:** Cumulative HBsAg seroclearance according to baseline HBsAg levels.

	HBsAg Seroclearance		Crude HR (95%CI)	Cumulative Incidence (95%CI)		*p* Value
	Yes	No		5 Years	10 Years	
N. of subjects (%)	17 (11.6)	129 (88.4)	-	3.0% (1.2–7.9%)	17.4% (10.8–27.6%)	-
HBsAg levels, n (%)						0.009
<100 IU/mL	11 (23.9)	35 (76.1)	3.89 (1.08–14.00)	7.0% (2.3–20.1%)	35.1% (20.9–54.8%)	
100–1000 IU/mL	3 (6.4)	44 (93.6)	0.87 (0.17–4.35)	0%	4.4% (0.6–27.1%)	
>1000 IU/ml	3 (5.7)	50 (94.3)	1.00 *	2.1% (0.3–23.9%)	10.1% (3.2–29.2%)	

HR, hazard ratio; 95% CI, 95% confidence interval. * Reference group.

**Table 3:** Analysis of predictive factors for HBsAg seroclearance.

	HBsAg Seroclearance		Crude HR (95%CI)	*p* Value	Adjusted HR ^+^ (95%CI)	*p* Value
	Yes (*n* = 17)	No (*n* = 129)				
Baseline HBsAg levels, n (%)				0.002		0.01
<100 IU/mL	11 (23.9)	35 (76.1)	4.18 (1.55–11.32)		3.53 (1.29–9.69)	
≥100 IU/mL	6 (6.0)	94 (94.0)	1.00 *		1.00 *	
Sex, n (%)				0.02		0.06
Male	13 (18.8)	56 (81.2)	3.51 (1.14–10.81)		2.84 (0.90–8.90)	
Female	4 (5.2)	73 (94.8)	1.00 *		1.00 *	
Age, mean ± SD, years	61.4 ± 7.7	57.2 ± 11.4	1.04 (0.99–1.09)	0.12	1.03 (0.98–1.09)	0.30
Baseline BMI, mean ± SD, Kg/m^2 **§**^	27.7 ± 4.0	26.2 ± 4.6	1.11 (0.98–1.25)	0.20		
Follow-up, mean ± SD, months	100.6 ± 31.6	85.3 ± 31.8	0.97 (0.94–1.00)	0.23		
Baseline HBV-DNA, median (IQR), IU/mL	310 (104–1043)	572 (164–1930)	1.00 (0.99–1.01)	0.78		
Baseline FibroScan, median (IQR), Kpa	4.3 (3.8–5.3)	4.9 (4.3–5.6)	0.86 (0.49–1.50)	0.78		
Liver steatosis, n/N (%) **^§^**	7/10 (70.0)	40/103 (38.8)	3.13 (0.81–12.13)	0.08	2.12 (0.54–8.37)	0.26

HR, hazard ratio; 95% CI, 95% confidence interval; BMI, body mass index. ^+^ Adjusted for baseline HBsAg level and sex. * Reference group. ^§^ Missing data: 32 patients for BMI; 33 patients for steatosis.

## Data Availability

The data presented in this study are available on request from the corresponding author. The data are not publicly available due to privacy.

## References

[1] European Association for the Study of the Liver (2017). EASL 2017 Clinical Practice Guidelines on the management of hepatitis B virus infection. J. Hepatol..

[2] Chu C.M., Liaw Y.F. (2010). Hepatitis B surface antigen seroclearance during chronic HBV infection. Antivir. Ther..

[3] Yeo Y.H., Tseng T.C., Hosaka T., Cunningham C., Fung J.Y.Y., Ho H.J., Kwak M.S., Trinh H.N., Ungtrakul T., Yu M.L. (2020). Incidence, Factors, and Patient-Level Data for Spontaneous HBsAg Seroclearance: A Cohort Study of 11,264 Patients. Clin. Transl. Gastroenterol..

[4] Brunetto M.R., Oliveri F., Colombatto P., Moriconi F., Ciccorossi P., Coco B., Romagnoli V., Cherubini B., Moscato G., Maina A.M. (2010). Hepatitis B surface antigen serum levels help to distinguish active from inactive hepatitis B virus genotype D carriers. Gastroenterology.

[5] Brouwer W.P., Chan H.L., Brunetto M.R., Martinot-Peignoux M., Arends P., Cornberg M., Cherubini B., Thompson A.J., Liaw Y.F., Marcellin P. (2016). Good Practice in using HBsAg in Chronic Hepatitis B Study Group (GPs-CHB Study Group). Repeated Measurements of Hepatitis B Surface Antigen Identify Carriers of Inactive HBV During Long-term Follow-up. Clin. Gastroenterol. Hepatol..

[6] Chu C.M., Hung S.J., Lin J., Tai D.I., Liaw Y.F. (2004). Natural history of hepatitis B e antigen to antibody seroconversion in patients with normal serum aminotransferase levels. Am. J. Med..

[7] Yoshida K., Enomoto M., Tamori A., Nishiguchi S., Kawada N. (2021). Combination of Entecavir or Tenofovir with Pegylated Interferon-α for Long-Term Reduction in Hepatitis B Surface Antigen Levels: Simultaneous, Sequential, or Add-on Combination Therapy. Int. J. Mol. Sci..

[8] European Association for the Study of the Liver (2012). EASL clinical practice guidelines: Management of chronic hepatitis B virus infection. J. Hepatol..

[9] Khetsuriani N., Mosina L., Van Damme P., Mozalevskis A., Datta S., Tohme R.A. (2021). Progress Toward Hepatitis B Control—World Health Organization European Region, 2016–2019. MMWR Morb. Mortal. Wkly. Rep..

[10] Mazzitelli M., Greco G., Serapide F., Scaglione V., Morrone H., Marascio N., Giancotti A., Liberto M., Matera G., Trecarichi E. (2021). Outcome of HBV screening and vaccination in a migrant population in southern Italy. Infez. Med..

[11] Hadziyannis S., Papatheodoridis G.V. (2006). Hepatitis B e antigen-negative chronic hepatitis: Natural history and treatment. Semin. Liver Dis..

[12] Zarski J.P., Marcellin P., Leroy V., Trepo C., Samuel D., Ganne-Carrie N., Barange K., Canva V., Doffoel M., Cales P. (2006). Characteristics of patients with chronic hepatitis B in France: Predominant frequency of HBe antigen negative cases. J. Hepatol..

[13] Tseng T.C., Liu C.J., Chen C.L., Yang H.C., Su T.H., Wang C.C., Yang W.T., Kuo S.F., Liu C.H., Chen P.J. (2013). Risk stratification of hepatocellular carcinoma in hepatitis B virus e antigen-negative carriers by combining viral biomarkers. J. Infect. Dis..

[14] Han Z.G., Qie Z.H., Qiao W.Z. (2016). HBsAg spontaneous seroclearance in a cohort of HBeAg-seronegative patients with chronic hepatitis B virus infection. J Med Virol..

[15] Chien T.L., Wang J.H., Kee K.M., Chen C.H., Hung C.H., Lu S.N. (2016). Factors Predicting HBsAg Seroclearance and Alanine Transaminase Elevation in HBeAg-Negative Hepatitis B Virus-Infected Patients with Persistently Normal Liver Function. PLoS ONE.

[16] Norder H., Couroucé A.M., Coursaget P., Echevarria J.M., Lee S.D., Mushahwar I.K., Robertson B.H., Locarnini S., Magnius L.O. (2004). Genetic diversity of Hepatitis B virus strains derived worldwide: Genotypes, subgenotypes and HBsAg subtypes. Intervirology.

[17] De Franchis R., Meucci G., Vecchi M., Tatarella M., Colombo M., Del Ninno E., Rumi M.G., Donato M.F., Ronchi G. (1993). The natural history of asymptomatic hepatitis B surface antigen carriers. Ann. Intern. Med..

[18] Manno M., Camma C., Schepis F., Bassi F., Gelmini R., Giannini F., Miselli F., Grottola A., Ferretti I., Vecchi C. (2004). Natural history of chronic HBV carriers in Northern Italy: Morbidity and mortality after 30 years. Gastroenterology.

